# Intranasal dopamine stabilizes prefrontal cortical CaMKIIα-positive neuronal population dynamics during object discrimination

**DOI:** 10.1038/s41598-026-65870-1

**Published:** 2026-08-06

**Authors:** Owen Y. Chao, Ezequiel Marron Fernandez de Velasco, Flávio Freitas Barbosa, Susanne Nikolaus, Joseph P. Huston, Yi-Mei Yang

**Affiliations:** 1 Graduate Institute of Brain and Mind Sciences, College of Medicine, National Taiwan University, Taipei, 100233 Taiwan; 2 Department of Biomedical Sciences, University of Minnesota Medical School, Duluth, MN 55812 USA; 3 Department of Pharmacology, University of Minnesota, Minneapolis, MN 55455 USA; 4 Department of Psychology, Federal University of Paraíba, João Pessoa, 58051 Brazil; 5 Clinic of Nuclear Medicine, University Hospital Düsseldorf, Heinrich Heine University of Düsseldorf, 40225 Düsseldorf, Germany; 6 Center for Behavioral Neuroscience, Heinrich Heine University of Düsseldorf, 40225 Düsseldorf, Germany; 7 Department of Neuroscience, University of Minnesota, Minneapolis, MN 55455 USA

**Keywords:** GCaMP, Schizophrenia, ADHD, Psychiatrics, Autism, Working memory, Neuroscience, Physiology

## Abstract

Dopamine (DA) is a key modulator of prefrontal cortex function, yet how distinct dopaminergic states shape the temporal organization of cortical population activity during behavior remains poorly understood. We combined in vivo fiber photometry of dorsomedial prefrontal cortex (dmPFC) putative glutamatergic neurons with an object-based attention (OBAT) task in male mice to examine this question. Intranasal DA (IN-DA), which accesses the brain directly via nose-to-brain pathways, left locomotion and exploratory behavior unchanged. Although both vehicle- and IN-DA- treated mice showed a preference for the novel object, this preference reached statistical significance only in the IN-DA group. IN-DA selectively amplified calcium activity within the familiar-object zone and enhanced movement-normalized calcium spike rates. Strikingly, IN-DA reduced both the mean and variability of calcium transient intervals, reflecting a shift toward more frequent and temporally regular events during object processing. To model the network consequences of supraphysiological dopaminergic drive, we administered the psychostimulant amphetamine as a proxy for dopaminergic excess. In contrast to the stabilizing effects of IN-DA, amphetamine induced locomotor hyperactivity accompanied by a collapse of temporal structure, characterized by prolonged inter-transient intervals and markedly increased variability. Together, these findings demonstrate that dopaminergic modulation shapes not only the magnitude but also the temporal organization of dmPFC population activity. At the network level, physiological DA promotes a temporally stable prefrontal state, whereas dopaminergic excess destabilizes it. This provides population-level evidence for the inverted-U relationship between DA signaling and prefrontal function.

**Supplementary Information:**

The online version contains supplementary material available at https://doi.org/10.1038/s41598-026-65870-1.

## Introduction

The prefrontal cortex (PFC) is critical for cognitive functions, including working memory, attention, decision-making, and behavioral flexibility^1–4^. PFC networks must rapidly adapt to task demands to support these functions^5,6^. Dopamine (DA) heavily modulates the computational capacity of the PFC. Optimal DA levels enhance neuronal signaling processing, whereas insufficient or excessive DA levels impairs network integrity, an inverted-U relationship well-documented across species^7–10^. Consistent with this framework, DA receptor activation can improve attention and working memory^11–13^, while psychostimulant-induced DA “overtone”, such as that produced by amphetamine, often disrupts these functions^14^. Dysregulated DA signaling is strongly implicated in psychiatric disorders including attention deficit-hyperactivity disorder (ADHD), schizophrenia, and autism^15–18^. However, it remains unclear how real-time population dynamics differ between an ‘optimized’ and a ‘hyper-dopaminergic’ state.

Mechanistically, DA enhances the ‘signal-to-noise’ ratio of PFC pyramidal neurons^9,19–21^ through modulation of intrinsic excitability and engagement of inhibitory interneurons^22–25^. Yet most physiological evidence comes from slice preparations or single-unit recordings, which may not capture the ensemble dynamics required for complex tasks. Complexity increases further: midbrain dopaminergic neurons co-release glutamate^26,27^, implicating additional modulatory influences on PFC circuits. It remains unclear whether optimal DA tone primarily modulates the magnitude of neural population activity or instead promotes more temporally organized patterns of ensemble activity that support information processing during task performance.

Studying these mechanisms in vivo has been challenged by limitations in DA delivery. Systemic administration of DA receptor agonists often produce widespread peripheral effects^28^, and DA itself poorly crosses the blood-brain barrier. Intranasal administration offers a non-invasive alternative that bypasses the blood-brain barrier^29^, allowing delivery of DA directly to the central nervous system^30^. This method models a physiologically relevant elevation of DA tone while minimizing systemic side effects. In rodents, intranasal DA (IN-DA) administration elevates extracellular DA levels in the dorsal striatum and nucleus accumbens^31^, modulates the activity of tyrosine hydroxylase, which catalyzes DA synthesis^32,33^, and shows therapeutic promise in models of Parkinson’s disease^34^, ADHD^32,35,36^, aging^37^, schizophrenia^38^ and autism^33^.

To dissect the population-level mechanisms of dopaminergic tuning, we employed in vivo fiber photometry to monitor calcium dynamics in the dorsomedial prefrontal cortex (dmPFC) of male mice. We compared two pharmacological states: (1) an “optimized” dopaminergic state, induced by IN-DA during an object-based attention task (OBAT), and (2) a “hyper-dopaminergic” state, induced by amphetamine during open-field behavior. Amphetamine-induced monoamine release served as a model of the dopaminergic excess associated with psychosis-like states^39,40^. By quantifying both the magnitude and temporal structure of dmPFC calcium activity, we aimed to identify population-level signatures that distinguish physiologically relevant dopaminergic tuning from the destabilizing effects of monoaminergic overtone on prefrontal networks.

## Materials and methods

### Subjects

C57BL/6J male mice, aged 8–12 weeks, were obtained from The Jackson Laboratory (Bar Harbor, ME. Stock No.000664). Animals were housed in groups of four to five per cage, with *ad libitum* access to water and food. They were maintained in a controlled environment with regulated temperature and humidity under a 12 h light/dark cycle (lights off at 19:00). All procedures were approved by the Institutional Animal Care and Use Committee (Protocol ID: 2206–40190 A) at the University of Minnesota, in accordance with National Institute of Health guidelines.

### Surgery

Mice were anesthetized using a ketamine and xylazine cocktail (100 mg/kg and 10 mg/kg, respectively, i.p.). The animal’s head was shaved and positioned in a stereotaxic frame (Stoelting, Wood Dale, IL). The skull was exposed, and the head was leveled to align bregma and lambda on the same horizontal plane. Holes were drilled above the dmPFC (AP: +2.0 mm; ML: ±0.3 mm, relative to the bregma). AAV8-CaMKIIα-jRCaMP1b (0.3–0.5 µl; titer: 2.1 × 10^13^ GC/ml; Viral Innovation Core, University of Minnesota) was then infused into the targeted region (DV: -2.2 mm, relative to the bregma) at a flow rate of 0.09 µl/min using a Hamilton syringe attached to a micro-injector (Stoelting, Wood Dale, IL). At least ten min post-infusion, the syringe was slowly retracted, and an optic fiber (⌀1.25 mm, 200 μm core, 0.22 NA, 2.0 mm length; RWD Life, San Diego, CA) was implanted into the dmPFC at a depth of -2.0 mm. Virus infusions and optic fiber implantations were performed unilaterally, either in the right or left hemisphere. Subsequently, the optic fiber was secured to the skull using screws and dental cement. The animal was administered carprofen (5 mg/kg, s.c.) as an analgesic and placed on a heating pad set at 37 °C to maintain its body temperature until fully awake. Behavioral testing was performed five weeks after the surgery.

### Drug administration

Prior to drug administration, animals were habituated to handling for three consecutive days. Dopamine hydrochloride (Sigma-Aldrich, St. Louis, MO. H8502) was prepared in a viscous castor oil-based formulation (MetP Pharma, Emmetten, Switzerland). Before each behavioral test, it was freshly mixed at a dose of 3 mg/kg in a volume of 10 µl, kept on ice, and protected from light. The mouse was gently restrained and the DA or vehicle mixture was intranasally delivered (5 µl per nostril for ~ 30 s) with a pipette (Eppendorf North America, Hauppauge, NY). Behavioral testing began 10 min post-administration. The dosage and timing of IN-DA were based on previous literature in mice^33,41^.

For the amphetamine experiment, d-amphetamine hemisulfate salt (Sigma-Aldrich, St. Louis, MO. A5880) was dissolved in saline and administered in a dose of 1 mg/kg intraperitoneally (see below).

### Behavioral testing

A polyvinyl chloride box (40 × 40 × 30 cm) was used to assess locomotor activity and object recognition. Objects were weighted to prevent displacement by the animal. They had different textures (smooth or rough), sizes (diameter 7–9 cm, height 14–17 cm), shapes (columns or irregular) and materials (plastic or glass). The open field was located in a sound attenuating room (< 40 dB) with dim lighting (~ 50 lx). The room had several distinct geometric figures on the walls serving as spatial cues. A camera was mounted 1.5 m above the apparatus and connected to a computer to track the animal automatically using ANY-maze software (Stoelting, Wood Dale, IL). Behavioral tests were conducted between 10:00 and 17:00. Ethanol (70%) was used to clean the open field after each trial.

**Fig. 1:**
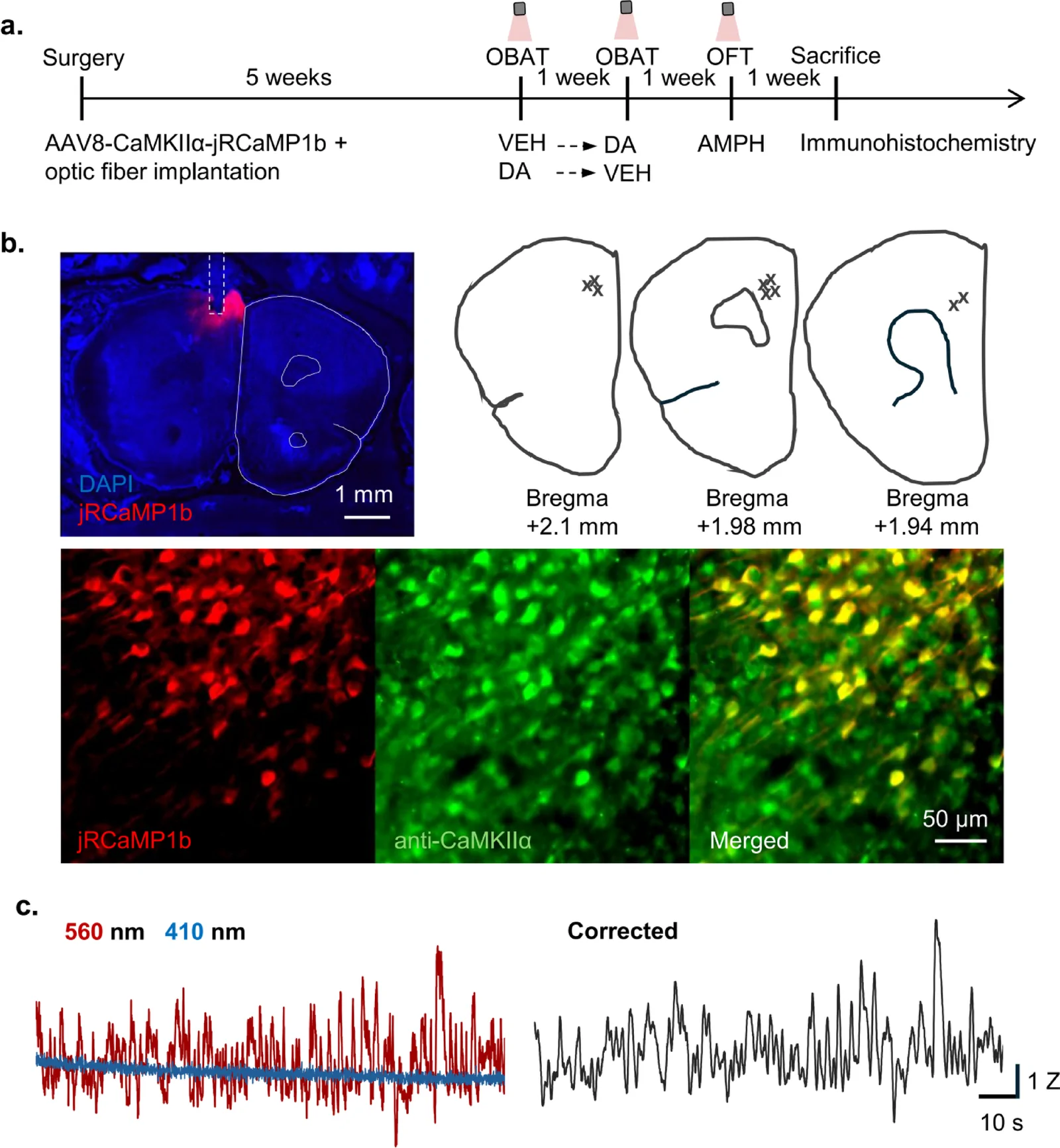
Experimental overview and calcium activity recording using fiber photometry. (**a**) Timeline of experimental procedures. Mice received AAV8-CaMKIIα-jRCaMP1b infusions and optic fiber implantation targeting the dorsomedial prefrontal cortex (dmPFC). They underwent the object-based attention test (OBAT) under vehicle (VEH) or dopamine (DA) treatment in a counterbalanced design, followed by the open field test (OFT) under amphetamine (AMPH) treatment. Their behaviors and calcium activity were recorded during the tests. Immunohistochemistry was performed after all experiments. (**b**) Locations of implanted fiber tips (crosses) within the dmPFC. An example showing the implanted optic fiber (indicated by dashed lines) and RCaMP1b expression in the dmPFC. Both the right and left implanted fiber tips are presented in one hemisphere. jRCaMP1b expression overlaps with anti-CaMKIIα–positive neurons (yellow). (**c**) Representative calcium traces recorded using in vivo fiber photometry with 410 nm (reference) and 560 nm (signal) excitation LEDs. The traces show the raw fluorescent signals (410 nm and 560 nm) and the corrected calcium signal after normalization of the signal channel to the reference channel.

For the IN-DA experiment, a within-subject counterbalanced design was applied to minimize individual variations in dmPFC calcium activity and behaviors. Half of the subjects received DA and the other half vehicle. One week later, treatments were switched, and the same behavioral paradigm was repeated. Another week later, the amphetamine experiment was conducted, with all subjects receiving amphetamine. The experimental timeline is shown in Fig. 1a.

Prior to each test, the optic cable of the fiber photometry system was connected to the animal’s head. The subject was then returned to its home cage to reduce the stress induced by this handling procedure. Calcium activities were monitored by the system (see below) to check the recording quality. Three to five min later, calcium signals were recorded during the behavioral trials. Repeated rotational behavior can cause twisting of the optic-fiber patch cable, resulting in mechanical artifacts characterized by rhythmic fluctuations in the fluorescence signals. Recordings exhibiting these artifacts were excluded from analysis (*n* = 2 in the amphetamine experiment).

The procedure of IN-DA fiber photometry experiment was: (1) the animal was administered vehicle or DA through its nostrils 10 min before testing; (2) the animal was returned to its home cage for 5 min; (3) the optic fiber was connected to the animal’s head; (4) the animal was returned to its home cage for several min; and (5) the behavioral testing was conducted.

#### Object-based attention test

Prior to testing, each animal was habituated to the open field. The paradigm consisted of a learning trial immediately followed by a test trial, with no delay between trials. Each trial had a duration of five min. During the learning trial, the animal was allowed to freely explore two distinct objects placed within the arena. For the test trial, one of the familiar objects was replaced with a novel object while the second object was replaced with an identical copy of one of the familiar objects, each positioned in the same location as in the learning phase. The animal remained inside the arena during the object replacement procedure. Object identity was counterbalanced across animals to minimize potential bias. Object exploration was defined as direct contacts with an object using the nose, head, or forepaws; contacts using the body or tail were not counted. Climbing on or sitting against the objects was also excluded. In addition to time spent exploring the objects, exploration bouts, locomotor activity (distance traveled), velocity (cm/s), time spent in the center zone, and time spent in movement versus rest were quantified. A discrimination index was also calculated as the time spent exploring the novel object minus the time spent exploring the old object, divided by total object time. Positive values indicate a preference for the novel object, whereas a value of 0 indicates no object preference. Animals exhibiting less than 10 s of total object exploration during the testing trial were excluded from all subsequent behavioral and calcium activity analyses (*n* = 2).

#### Open field test

The animal was connected to the fiber photometry setup and placed in the open field, where spontaneous locomotor activity and calcium fluorescence signals were recorded for 10 min. The 5–10 min recording served as a baseline. Immediately afterward, amphetamine (1 mg/kg, i.p.) was administered, and the animal was returned to the arena for an additional 50 min session during which distance traveled and calcium activity were monitored.

### Fiber photometry recording and analysis

The R820 multichannel fiber photometry system (RWD Life, San Diego, CA) was employed to record calcium activity, utilizing excitation light sources at wavelengths of 410 nm and 560 nm. This system generates analog outputs to drive the light sources with an alternation pattern of 60 Hz. The system operates using time-division multiplexed (interleaved) illumination, whereby the 560 nm and 410 nm excitation sources are sequentially activated in alternating acquisition frames. No sinusoidal frequency modulation or lock-in amplification is applied. Accordingly, the 60 Hz represents the internal CMOS camera frame acquisition rate rather than the effective sampling rate of each fluorescence channel. Because the two excitation channels are acquired alternately, the effective sampling rate for both the 560 nm signal channel and the 410 nm reference channel is 30 Hz. The 560 light source was dedicated to measuring jRCaMP1b calcium-dependent activity (signal channel), whereas the 410 nm light source served as the calcium-independent reference signal. To control background noise and movement artifacts, the jRCaMP1b signal was calibrated against the reference signal. Illumination power was adjusted at the tip of the optic fiber to 0.01–0.02 mW/mm^2^ for the reference channel and 0.04–0.06 mW/mm^2^ for the signal channel.

During behavioral assessments, calcium fluctuations were quantified by aligning the 560 nm signal (jRCaMP1b) with the 410 nm signal (reference) through a least-square linear fit. The relative fluorescence change (ΔF/F) was computed as (jRCaMP1b signal – fitted reference signal) / (fitted reference signal), and z-scores were calculated as (ΔF/F – mean) / standard deviation. The integrated software of the fiber photometry system automatically identifies calcium spikes, applying criteria that exceed 1 median absolute deviation with a minimum duration of 1 s. Calcium amplitudes (z-scores), the number of spikes and inter-spike intervals (ISI) were calculated and the coefficient of variation (CV) of spike amplitude and ISI were computed. CV represents the relative variability of a given dataset by calculating the ratio of the standard deviation to the mean ([standard deviation / mean] x 100). In this context, ISI is defined as the time interval between the peaks of consecutive calcium transients, representing the temporal structure of synchronized population bursts rather than single-unit discharge.

Spatial localization of calcium activity was analyzed by mapping calcium signals to the animal’s position within the testing arena. Z-score maps and spike maps were generated to visualize the spatial distribution of calcium signal intensity and calcium spike occurrences, respectively. Representative calcium traces and spike maps from a single animal are presented to illustrate the spatial patterns of neuronal activity. We defined 15 × 15 cm virtual zones for each object, providing at least a 6 cm boundary or exploration, and used these regions as object exploration zones for the spatial calcium activity analyses. The non-object area was defined as all areas outside the object zones. The map employs color coding from a spectrum of blue to red to represent z-scores ranging from − 4 to 4, with blue and red indicating negative and positive z-scores, respectively.

To visualize the relationship between calcium activity and locomotor speed, the two datasets were aligned by velocity, allowing calcium activity to be analyzed across a spectrum of motor activity (0–30 cm/s). Specifically, the animal’s instantaneous velocity was categorized into sequential 5 cm/s bins spanning 0 to > 30 cm/s. For each individual subject, the mean calcium z-score was calculated using only the time points falling within each velocity bin. The resulting values were subsequently averaged across animals to generate the group-level velocity-dependent calcium activity profiles.

Peri-event analysis was performed by generating time-aligned histograms within a 6-s window centered on behavioral onset. Behavioral onset was defined as either movement initiation or entry into predefined zones (areas adjacent to objects). Events not involving direct exploration of objects for at least 1 s were excluded (e.g., entering the zone while orienting elsewhere). Calcium signals were z-score normalized using the − 3 to 0 s pre-event period as baseline. Area-under-the-curve (AUC) analysis was used to determine whether calcium activity was significantly elevated relative to baseline.

To analyze fundamental features of population events, the density and cumulative probability distributions of transient amplitudes and ISI were plotted using all the identified calcium spikes. Density estimates were computed using kernel density estimation with a Gaussian kernel and bandwidth selection according to Scott’s rule (bw = n^−1/5^·σ; bw: the bandwidth of the kernel; n: the number of data points used for estimation; σ: the standard deviation of the data).

To attenuate photo-bleaching during the 60 min monitoring in the amphetamine experiment, we used an alternating recording protocol (20s ON / 20s OFF). The other settings remained the same as described.

### Immunohistochemistry and confocal imaging

After completion of behavioral testing, the animal was deeply anesthetized by ketamine (300 mg/kg. i.p.) and perfused transcardially with ice-cold phosphate-buffered saline (PBS, pH 7.4), followed by 4% paraformaldehyde. The brain was removed and immersed in 4% paraformaldehyde overnight at 4 °C, and then stored in a 30% sucrose PBS solution at 4 °C until it sank. The brain was sectioned with a vibratome (Leica VT1200S, Buffalo Grove, IL) and the dmPFC slices with a thickness of 50 μm were collected at 200 μm intervals resulting in 5–6 slices per brain. For immunofluorescence, the slices were washed with PBS three times and incubated in a blocking solution (2% goat serum and 0.2% Triton X-100 in PBS) with a rabbit anti-CaMKIIα antibody (1:200; PA5-99558, Invitrogen, Waltham, MA) overnight at 4 °C. The slices were washed in PBS three times and incubated in goat anti-rabbit Cy5 (1:500; A10523, Invitrogen, Waltham, MA) for 3 h at room temperature. They were then washed in PBS three times and cover-slipped with the Vectashield mounting medium with DAPI (H-1500, Vector Laboratories, Newark, CA).

Imaging was performed using a Nikon AX-R confocal microscope (Nikon, Melville, NY) with 20x objectives. The location of the implanted optic fiber was imaged and identified based on the mouse brain atlas^42^. For images taken, 25–30 z-steps of 0.5 μm intervals were acquired. The images were stacked together and the brightness and contrast were adjusted. The jRCaMP1b-expressing neurons were considered as excitatory neurons if their expression overlapped with the anti-CaMKIIα signal.

### Statistics

OBAT behavioral and calcium activity data were analyzed using repeated two-way ANOVAs with the appropriate within-subject factors for each analysis. Amphetamine-induced open-field behavior and calcium activity were analyzed using repeated one-way ANOVA across time bins. One-sample *t*-tests (against zero) were performed to determine whether values significantly differed from baseline. Kolmogorov–Smirnov tests were used to compare density and cumulative probability distributions between treatments. Post hoc pairwise comparisons were corrected using the Holm–Bonferroni method, whereas multiple comparisons against a single control group were corrected using Dunnett’s test. All tests were two-tailed, with statistical significance set as *p* < 0.05. Results were presented as mean ± SEM with data points representing the individual animals.

## Results

### Recording calcium activity from prefrontal cortical neurons in mice

To examine how DA modulates the prefrontal networks, we expressed the red fluorescent calcium indicator jRCaMP1b in the mouse dmPFC by infusing AAV8-CaMKIIα-jRCaMP1b, followed by implantation of an optic fiber in the infusion site. The CaMKIIα promoter helps preferential expression in glutamatergic neurons. jRCaMP1b was selected for its red-shifted emission spectrum, which reduces tissue autofluorescence and improves signal-to-noise ratio^43^. Optic fibers were targeted to the dmPFC, including the anterior cingulate and prelimbic cortices, approximately 2.0 ± 0.1 mm anterior to the bregma (Fig. 1b). One animal was excluded from analysis due to incorrect fiber placement.

Histological verification was performed using immunohistochemistry to evaluate viral expression within the dmPFC. The results showed that the jRCaMP1b-expressing neurons co-labeled with CaMKIIα (Fig. 1b). Because glutamatergic pyramidal neurons constitute the predominant neuronal population in the mouse PFC, whereas GABAergic interneurons account for approximately 20% ^44^, the recorded bulk calcium signals are expected to be largely driven by excitatory network activity. Nevertheless, recent evidence indicates that viral CaMKIIα promoter constructs can exhibit limited expression in subsets of GABAergic interneurons^45^. Therefore, fiber photometry signals obtained in the present study should be interpreted as reflecting predominantly, but not exclusively, glutamatergic neuronal population activity.

During behavior, calcium-dependent fluorescence signals from jRCaMP1b-expressing neurons were recorded in vivo using fiber photometry. Excitation at 560 nm captured calcium-dependent fluorescence, whereas a 410 nm isosbestic wavelength served as a calcium-independent reference for motion and photobleaching correction. This configuration produced stable, high-quality recordings, with 560 nm signals tightly aligned to the 410 nm reference (Fig. 1c). Calcium transients were automatically detected and quantified using the photometry system’s analysis software.

### Intranasal dopamine administration preserves object discrimination behavior while selectively amplifying prefrontal calcium responses in the familiar object zone

During the learning trial of the OBAT, IN-DA did not change distance traveled, center time, exploration bouts, total object exploration, or time spent moving and resting (*ps* > 0.05), indicating no baseline behavioral differences between treatments.

In the testing trial, a repeated two-way ANOVA revealed a significant main effect of “object” (F_1,6_=8.149, *p* = 0.029), but no effect of “treatment” (F_1,6_=0.037, *p* = 0.853) or “treatment x object” (F_1,6_=0, *p* = 1) on exploration bouts. Likewise, a main effect of “object” (F_1,6_=10.067, *p* = 0.019), but not “treatment” (F_1,6_=2.202, *p* = 0.188) and “treatment x object” (F_1,6_=0.232, *p* = 0.647), was found in the analysis of object exploration time. These results demonstrate that overall, animals discriminated the novel object from the old one regardless of treatments.

**Fig. 2:**
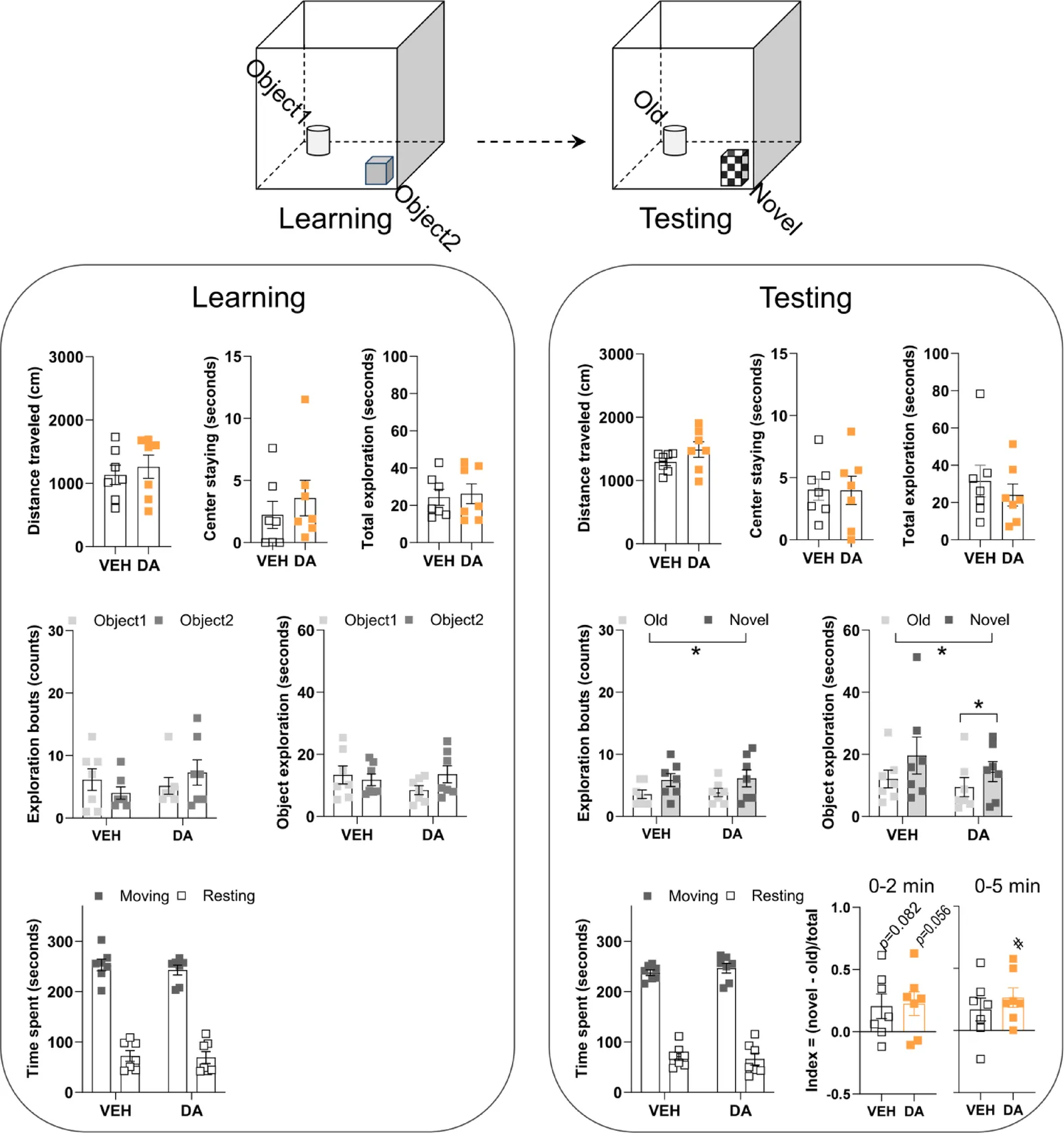
Dopamine effects on object-based attention performance. Dopamine (DA) did not affect behavior during the learning trial, in which mice were exposed to two objects, compared with vehicle (VEH). During the testing trial, both treatment groups explored the novel object more than the old one. DA stabilizes the preference for the novel object without affecting total distance traveled, center time, total object exploration time, or durations spent moving or resting, compared with VEH. Data shown as mean ± SEM with individual points. Significant effects (*p* < 0.05) were determined by ANOVA or by paired *t*-tests with Holm-Bonferroni adjusted *p* values. # *p* < 0.05, one-sample t-tests compared with 0.

To examine object exploration patterns within each treatment condition, exploratory paired *t*-tests comparing exploration times of the old and novel objects were performed separately for each treatment. Following Holm-Bonferroni corrections, IN-DA-treated mice spent significantly more time exploring the novel object than the old one (t_6_ = 2.481, *p* = 0.048, Cohen’s d = 2.32), whereas vehicle-treated mice failed to reach the significant novelty preference (t_6_ = 1.781, *p* = 0.125, Cohen’s d = 1.22). Consistently, a significantly positive discrimination index was identified within the IN-DA treatment (t_6_ = 3.406, *p* = 0.014, Cohen’s d = 0.21) but not in the vehicle treatment (t_6_ = 1.844, *p* = 0.115, Cohen’s d = 0.24). Given that the first 2 min represents the most sensitive window for object exploration^46^, we isolated and analyzed the discrimination index during this time phase. During the first 2 min, both treatment groups demonstrated a positive trend compared to 0 (t_6_ = 2.084, *p* = 0.082, Cohen’s d = 0.26 for vehicle; t_6_ = 2.368, *p* = 0.056, Cohen’s d = 0.25 for IN-DA). This suggests that the current experimental procedure (e.g., IN administration or fiber-optic tethering) may affect the initial exploration, whereas the novelty preference was sustained during the late phase following IN-DA. However, direct comparisons between treatments did not reveal a significant treatment effect on the discrimination index (*p*s > 0.05). No other measures differed between treatments (*ps* > 0.05) (Fig. 2), indicating that IN-DA did not affect locomotion (distance traveled), anxiety-like behavior (center time), or motivation (total object exploration time).

**Fig. 3:**
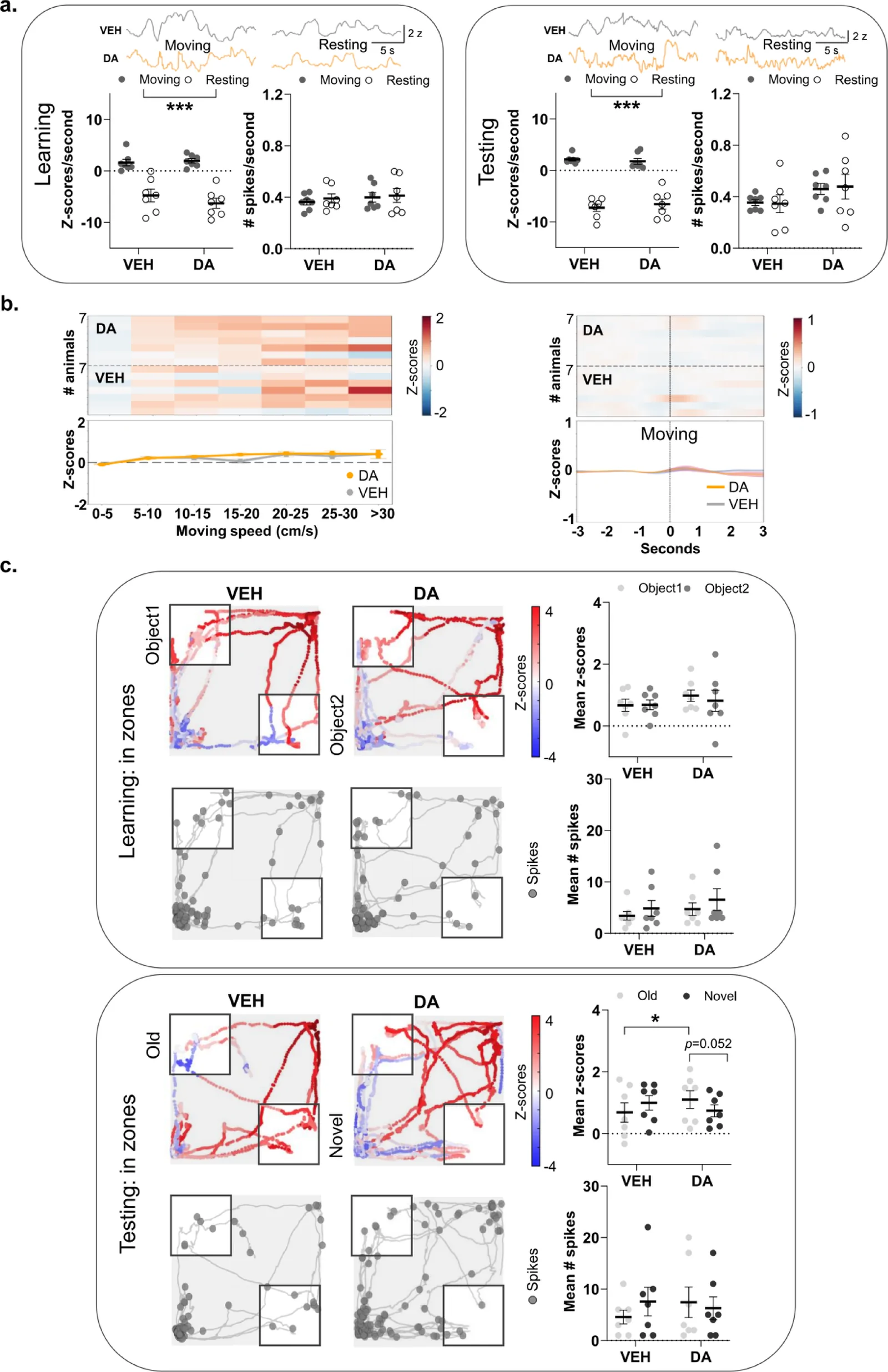
Dopamine increases calcium responses in the old object zone in the object attention task. (**a**) Mean calcium signal (z-scores per second), but not spike rate, was higher during movement than rest in both learning and testing trials for both treatments. (**b**) Calcium activity did not correlate with moving speed or movement onset. Top panels show trial-aligned calcium traces from individual subjects (each row represents one subject). Bottom panels show average moving speed (from 0 to > 30 cm/s) or a 6-s time window aligned to movement onset (time 0). Gray and orange lines indicate mean vehicle and dopamine calcium traces. (**c**) Exemplar heatmaps of calcium activity across spatial locations from a single animal. Squares indicate zones of object placement. In the learning trial, z-scores and spike counts did not differ between treatments. In the testing trial, dopamine increased mean z-scores, but not spike counts, in the old object zone relative to vehicle. Data shown as mean ± SEM with individual points. Significant effects (*p* < 0.05) were determined by paired *t*-tests with Holm-Bonferroni adjusted *p* values.

To characterize dmPFC activity, we quantified z-scores per second and transient frequency (spike counts) per second during movement and rest. For z-scores, significant effects of “state” (F_1,6_=43.46, *p* < 0.001 and F_1,6_=112.481, *p* < 0.001 for learning and testing trials, respectively), but not “treatment” (F_1,6_=0.83, *p* = 0.398 and F_1,6_=0.83, *p* = 0.784 for learning and testing trials, respectively) and “treatment x state” (F_1,6_=0.785, *p* = 0.41 and F_1,6_=0.59, *p* = 0.472 for learning and testing trials, respectively), were found in both trials. Thus, z-scores were significantly higher during movement than resting across trials and treatments. No significant effects were found regarding transient frequency and treatments (*ps* > 0.05) (Fig. 3a). dmPFC putative glutamatergic neurons thus encoded behavioral state primarily through calcium amplitude, not event rate.

To determine whether calcium activity reflects movement per se, we examined mean z-scores across the continuous range of locomotor velocities (0 to > 30 cm/s). Although activity was state-dependent (higher during movement than rest), we found no linear amplitude–velocity relationship within the movement periods. Event-aligned analysis triggered on movement onset similarly revealed no significant calcium elevations (*ps* > 0.05), and no treatment effects were observed (*p* > 0.05) (Fig. 3b). Thus, while calcium amplitudes were generally elevated during the active state, they were not linearly driven by velocity magnitude or movement initiation. dmPFC putative glutamatergic activity thus likely reflects cognitive rather than motor processing.

We next analyzed spatial patterns of calcium activity, focusing on the object zones where object exploration occurred. Given that individual exploration bouts are often brief and highly variable in duration and frequency across animals (Fig. 2), we analyzed calcium activity within the zones to robustly capture neural dynamics during object-directed exploration. In the learning trial, when animals first encountered the objects, they explored them relatively equally and exhibited comparable calcium amplitudes and transient frequencies across the two object zones, with no treatment effects (*ps* > 0.05). In contrast, during the testing trial, a significant “treatment x zone” (F_1,6_=6.606, *p* = 0.042), but not “treatment” (F_1,6_=0.2, *p* = 0.67) or “zone” (F_1,6_=0.015, *p* = 0.905), effect was found. As the interaction effect was identified, multiple comparisons were conducted with Holm-Bonferroni correction. IN-DA significantly increased calcium amplitudes within the old object zone compared with vehicle (t_6_ = 2.510, *p* = 0.046, Cohen’s d = 0.44), with a trend toward higher amplitudes relative to the novel object zone within the IN-DA treatment (t_6_ = 2.417, *p* = 0.052, Cohen’s d = 0.39). No treatment differences were found in transient frequency (*ps* > 0.05) (Fig. 3c).

To examine whether object-zone modulations were spatially specific, we also analyzed calcium dynamics in the non-object area. Although the non-object area exhibited a higher transient frequency, these events were characterized by significantly lower mean calcium amplitudes compared to object zones (**Supplementary Fig. 1**). This indicates that while dmPFC exhibits continuous baseline firing during general arena navigation, object exploration recruits high-amplitude calcium responses (Fig. 3c). Furthermore, during testing, IN-DA elevated transient frequency in non-object areas while selectively boosting amplitude within the old object zone, consistent with the interaction effects observed (**Supplementary Fig. 1**). These results demonstrate that DA selectively enhanced calcium amplitude in the old object zone, a modulation that may underlie the observed effect (Fig. 2).

### Intranasal dopamine administration increased calcium rates after normalizing for movement distance and duration

**Fig. 4:**
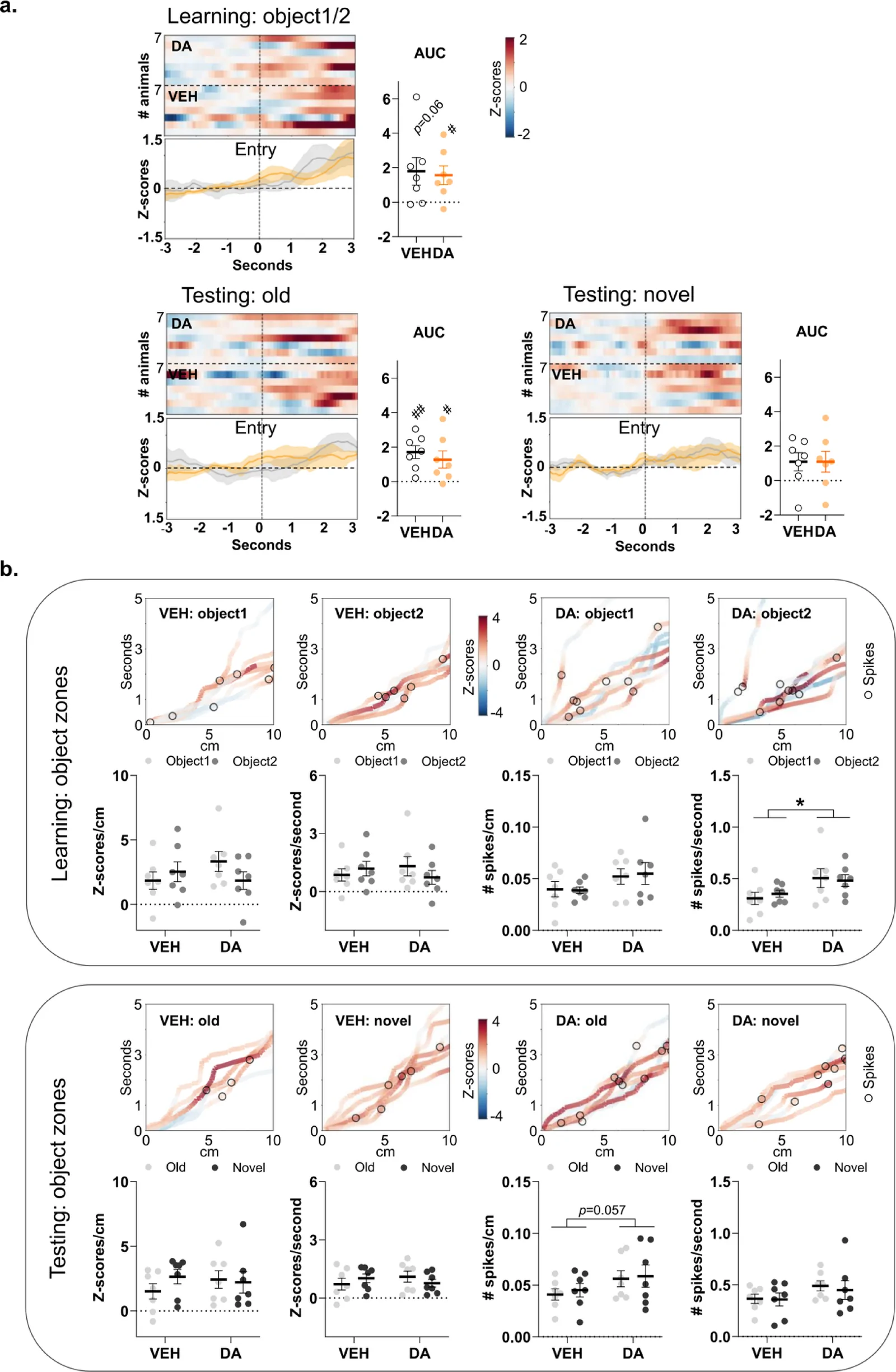
Dopamine increases normalized calcium spike counts. (**a**) Calcium responses aligned to zone entry. Entering the object1/2 and old zones was associated with elevated calcium activity in both treatment conditions, quantified using area under the curve (AUC). Top panels show calcium traces for individual mice (each row represents one subject), and bottom panels show trial-aligned averages within a 6-s window centered on zone entry (time 0). Gray and orange lines represent vehicle (VEH) and dopamine (DA) groups, with SEM shading. (**b**) Calcium z-scores and spike counts within each zone were normalized by moving distance and time spent in that zone. Representative calcium activity traces are shown together with their distance traveled (cm) and movement duration (seconds). DA treatment increased the normalized number of calcium spikes compared with VEH. Data shown as mean ± SEM with individual points. Significant effects (*p* < 0.05) are indicated by ANOVAs. # *p* < 0.05, ## *p* < 0.01, one-sample *t*-tests compared to 0.

Because raw calcium traces exhibited substantial baseline variability across trials and subjects (**Supplementary Fig. 2**), signals were normalized to a pre-entry baseline window (-3 to 0s) to isolate event-related responses. When calcium signals were time-locked to entries into the object zones, z-scores increased upon object engagement during the learning trial for both groups (one-sample *t*-tests: vehicle: t_6_ = 2.229, *p* = 0.067, marginal, Cohen’s d = 2.13; IN-DA: t_6_ = 2.873, *p* = 0.028, Cohen’s d = 1.44). In the testing trial, both treatment groups exhibited significant elevations in calcium activity within the old object zone (one-sample *t*-tests: vehicle: t_6_ = 4.553, *p* = 0.004, Cohen’s d = 0.99; IN-DA: t_6_ = 2.554, *p* = 0.043, Cohen’s d = 1.32), but not in the novel object zone (*ps* > 0.05) (Fig. 4a). These results indicate that dmPFC putative glutamatergic neurons responded to object-related information, including both initial novelty (learning trial) and familiar-object processing (testing trial).

To further control for locomotor confounds (Fig. 3a), we normalized calcium activity (z-scores and transient/spike counts) to distance traveled and time spent moving within the defined zones. During the learning trial, analysis of normalized spike number per second revealed a significant effect of “treatment” (F_1,6_=6.493, *p* = 0.044), but no effects of “zone” (F_1,6_=0.081, *p* = 0.785) or “treatment x zone” (F_1,6_=0.863, *p* = 0.389), indicating increased calcium spike activity following IN-DA. During the testing trial, analysis of spike number per cm revealed a trend toward a main effect of “treatment” (F_1,6_=5.501, *p* = 0.057), with no effect of “zone” (F_1,6_=1.494, *p* = 0.267) or “treatment x zone” (F_1,6_=0.085, *p* = 0.781). No significant treatment effects were detected for the remaining measures (*ps* > 0.05) (Fig. 4b). These findings suggest that IN-DA increases normalized dmPFC calcium spiking in object exploration-related activity.

### Intranasal dopamine administration reduced inter-transient interval variability during the testing trial

**Fig. 5:**
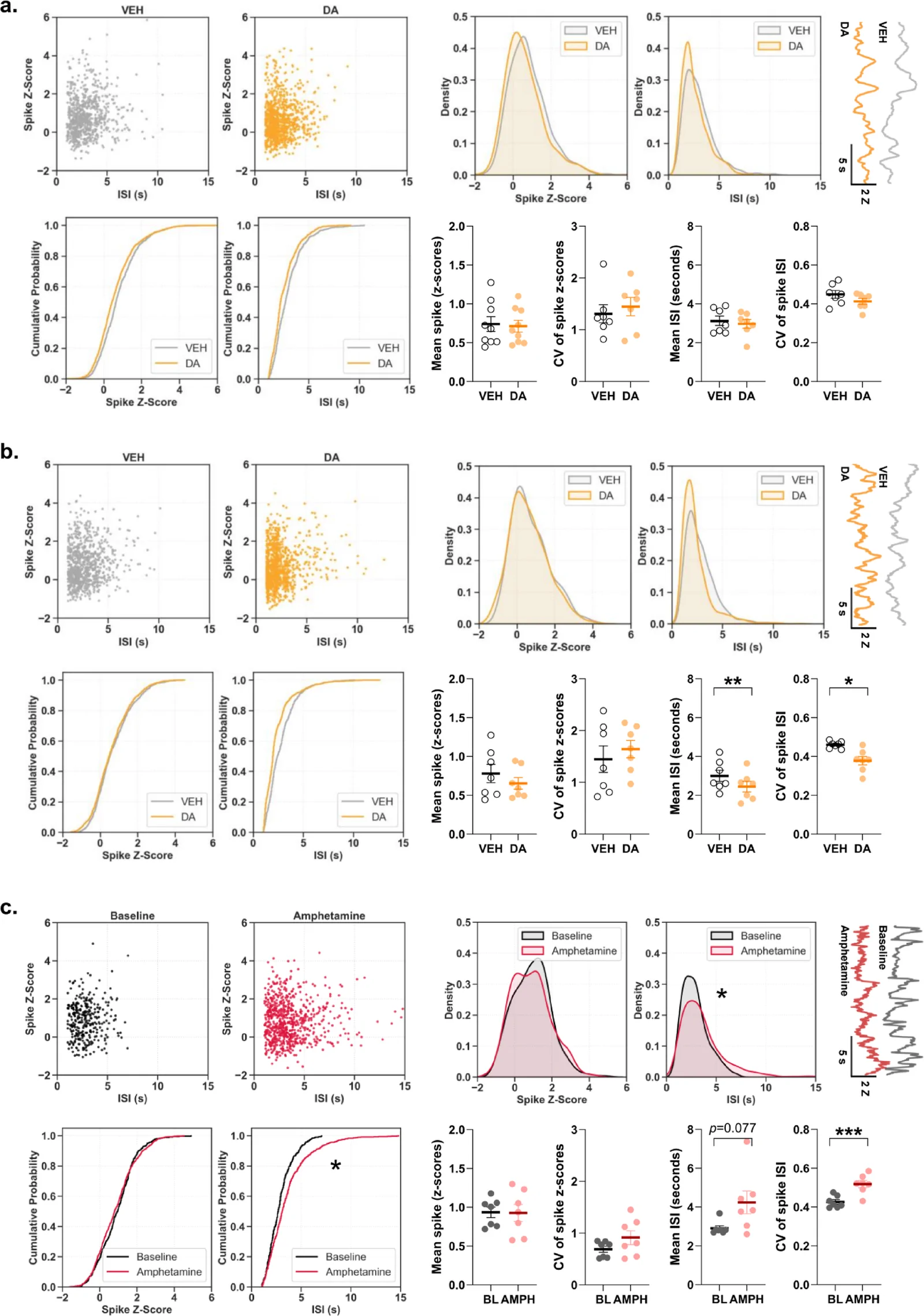
Dopamine alters variability of calcium dynamics. (**a**) In the learning trial of the object-based attention test (OBAT), dopamine (DA) did not change calcium activity properties relative to vehicle (VEH). (**b**) In the testing trial of OBAT, overall calcium activity properties were similar between treatments, but DA reduced the mean spike inter-spike intervals (ISI), as well as the coefficient of variation (CV) of ISI. (**c**) Amphetamine (AMPH) increased ISI duration and elevated CV compared with baseline in the open field test. Data shown as mean ± SEM with individual values. Statistical significance (*p < 0.05, **p < 0.01, ***p < 0.001) is based on paired *t*-tests or Kolmogorov-Smirnov tests.

Finally, we assessed whether IN-DA altered fundamental features of dmPFC calcium signaling during the OBAT. In the learning trial, no treatment effects were detected across multiple measures, including transient amplitudes and ISI scatter distributions, density plots, cumulative probability curves, or summary metrics such as mean transient amplitude, mean ISI, or their variability (*ps* > 0.05) (Fig. 5a). Thus, IN-DA did not influence baseline calcium dynamics during initial object exploration.

During the testing trial, most calcium parameters again did not differ between treatments (*ps* > 0.05). However, IN-DA significantly reduced ISI (t_6_ = 4.188, *p* = 0.006, Cohen’s d = 0.35) and its variability (t_6_ = 3.274, *p* = 0.017, Cohen’s d = 0.17) (Fig. 5b), indicating more frequent and temporally regular calcium events in dmPFC putative glutamatergic neurons following IN-DA during the cognitively demanding session.

### Amphetamine induced hyperactivity and increased inter-transient interval variability in the open field test

To determine whether the reduced ISI variability observed after IN-DA was maintained under conditions of supraphysiological drive, we administered the psychostimulant amphetamine to the same animals and assessed dmPFC calcium activity during the open field test. This open-field assay probed dmPFC signaling during locomotor hyperactivity and monoaminergic overtone, modeling the network instability at the “hyper-dopaminergic” end of the inverted-U curve, distinct from the organized attentional demands of the OBAT.

Amphetamine was administered 10 min after the open field test, and recordings continued for an additional 50 min (total recording for 60 min). As expected, amphetamine significantly increased locomotor activity (F_11,88_=4.433, *p* < 0.001). Distance traveled rose significantly beginning 20 min post-administration and remained elevated almost until the end of the session relative to baseline (Table 1). We then assessed whether amphetamine changed core features of dmPFC calcium dynamics. Amphetamine produced a markedly more dispersed ISI distribution (Fig. 5c), accompanied by a right-shifted ISI density function and a prolonged ISI cumulative probability curve (Kolmogorov-Smirnov test: D = 0.159, *p* < 0.001). Furthermore, amphetamine substantially increased ISI variability (t_6_ = 6.663, *p* < 0.001, Cohen’s d = 0.14) and showed a trend toward increasing ISI (t_6_ = 2.13, *p* = 0.077, Cohen’s d = 1.66), while leaving other measures unchanged (*ps* > 0.05; Table 2).

**Table 1:** Distance traveled in the open field test. Repeated one-way ANOVA followed by paired *t*-tests compared to baseline. Values are presented as mean ± SEM.

Time bins	Distance traveled (cm)	Compare to baseline
0–5 min	378.05 ± 51.63	
5–10 min	377.65 ± 27.41	Baseline
Amphetamine administration		
10–15 min	391.93 ± 45.02	
15–20 min	583.64 ± 94.21	
20–25 min	624.30 ± 130.70	
25–30 min	513.53 ± 103.91	
30–35 min	825.34 ± 173.20	t_8_=2.698, *p* = 0.027
35–40 min	744.83 ± 140.73	t_8_=2.752, *p* = 0.025
40–45 min	907.70 ± 217.25	t_8_=2.483, *p* = 0.038
45–50 min	933.61 ± 195.16	t_8_=2.945, *p* = 0.019
50–55 min	820.21 ± 189.49	t_8_=2.421, *p* = 0.042
55–60 min	774.64 ± 197.11	

**Table 2:** Calcium spike measured in the amphetamine-open field test. Repeated one-way ANOVAs revealed no significant effects of “time bins” (number of spikes: F_11,66_=1.127, *p* = 0.356; mean spike z-scores: F_11,66_=0.25, *p* = 0.992). Values are presented as mean ± SEM.

Time bins	Number of spikes	Mean spike z-scores
0–5 min	12.14 ± 1.39	1.86 ± 0.14
5–10 min	21.86 ± 1.49	1.78 ± 0.09
Amphetamine administration		
10–15 min	15.29 ± 3.72	1.84 ± 0.17
15–20 min	17.00 ± 3.22	1.77 ± 0.11
20–25 min	20.14 ± 3.38	1.81 ± 0.10
25–30 min	14.00 ± 3.11	1.83 ± 0.13
30–35 min	15.14 ± 3.84	1.96 ± 0.19
35–40 min	18.71 ± 2.54	1.76 ± 0.04
40–45 min	15.00 ± 1.79	1.83 ± 0.20
45–50 min	16.57 ± 3.87	1.94 ± 0.09
50–55 min	20.00 ± 3.48	1.83 ± 0.13
55–60 min	13.57 ± 2.40	1.84 ± 0.08

These findings indicate that excessive dopaminergic tone disrupted the temporal organization of dmPFC putative glutamatergic activity: ISI became slower and more variable, and population event timing less reliable, during hyper-locomotion.

## Discussion

The present study demonstrates that dopaminergic state shapes the temporal organization of dmPFC population activity, not only its overall magnitude. During object-based attention, IN-DA selectively enhanced calcium responses within the familiar-object zone and reduced both the ISI and ISI variability, reflecting more frequent and temporally regular calcium events in dmPFC putative glutamatergic neurons. In contrast, amphetamine induced locomotor hyperactivity and disrupted this temporal structure, increasing both ISI and its variability. These findings identify temporal regularity of prefrontal population activity as a sensitive index of dopaminergic state and provide circuit-level evidence that physiological versus supraphysiological DA differentially regulates dmPFC network dynamics, consistent with the inverted-U relationship between DA signaling and prefrontal function.

Behaviorally, both vehicle- and IN-DA-treated mice exhibited a preference for the novel object, yet this preference reached statistical significance only in the IN-DA group (Fig. 2). This asymmetry suggests that IN-DA may stabilize or reinforce the familiar-object memory trace, facilitating more reliable discrimination without altering overall exploratory behavior.

The selective enhancement of calcium amplitudes within the old object zone (Fig. 3c) indicates that IN-DA facilitated the maintenance of familiar representations, a process critical for successful object recognition^47,48^. The interaction between dopaminergic systems and PFC is essential for the performance of object recognition memory^49^. The results are consistent with clinical studies on humans^50–52^ and rodents^11–13^, which showed improved attention and task performance following DA receptor agonist administration. Although the PFC is well-known for its role in novelty processing^53^; Fig. 4a), effective discrimination of a novel stimulus fundamentally relies on the stability of the pre-existing memory trace of the familiar object. Our data show that IN-DA did not globally increase dmPFC excitability, but amplified calcium signals when animals engaged with the familiar stimulus. This selective tuning is consistent with the signal-to-noise framework of dopaminergic modulation, in which optimal DA levels enhance task-relevant ensemble activity while suppressing background fluctuations^9,19–21^.

This increase persisted after normalizing calcium activity to movement distance and duration (Fig. 4b), indicating that it reflects intrinsic modulation of dmPFC activity rather than nonspecific locomotor arousal. IN-DA increased normalized calcium spike frequency during the learning (and likely the testing) phase, consistent with the concurrent reduction in ISI mean and variability (Fig. 5b). These findings indicate that dopaminergic enhancement restructured the temporal organization of dmPFC population activity. We propose that DA promotes a more temporally coherent pattern of ensemble firing during object learning and memory processing, a mechanism that may underlie the observed performance, though the precise relationship between these population dynamics and behavior awaits future investigation.

Beyond changes in response magnitude, our findings reveal a critical role for DA in regulating the temporal structure of prefrontal activity. IN-DA administration significantly reduced ISI and its variability during the testing trial (Fig. 5b), indicating a shift toward a more temporally stable pattern of population events. High temporal variability, often manifesting as irregular spike timing or stochastic jitter, can compromise the precision of neural coding and introduce noise into the network^54^. By attenuating this variability, DA acts as a “stabilizer”, promoting a more consistent temporal structure of glutamatergic activity that supports sustained information maintenance during cognitive tasks.

This stabilization can arise from the synergistic modulation of intrinsic and synaptic currents. DA, acting through D1 receptors, has been shown to potentiate slowly inactivating NMDA receptor-mediated currents, thereby broadening the temporal window for synaptic integration and supporting sustained depolarized “up-states” ^55,56^. Concurrently, DA modulates voltage-gated Na⁺ and K⁺ channels to regulate intrinsic excitability, reducing the first ISI and sharpening the precision of spike initiation^57^. Together, these cellular mechanisms likely manifest at the population level as the regular, high-amplitude calcium transients observed here, preventing stochastic degradation of information during cognitive processing.

In contrast to IN-DA, amphetamine induced pronounced network disruption, characterized by prolonged ISI and increased temporal variability. This aligns with the inverted-U model of dopaminergic modulation, in which excessive DA drives prefrontal circuits beyond their optimal dynamic range, leading to functional impairment^9,58,59^. Such suppression of glutamatergic output may arise from enhanced feed-forward inhibition: elevated DA levels preferentially increase the excitability of parvalbumin interneurons^25^, thereby dampening pyramidal neuron activity (prolonged ISI; Fig. 5c). Notably, the PFC is particularly sensitive to dopaminergic imbalance, as tonic (~ 5 Hz) and phasic (> 20 Hz) DA firing modes differentially engage prefrontal networks^27,60^. Furthermore, context-dependent chemogenetic manipulation of midbrain DA neurons bidirectionally modulates PFC calcium dynamics^61^. Importantly, amphetamine altered transient timing and variability without affecting calcium amplitudes, suggesting DA regulates dmPFC activity primarily through frequency- and timing-based neural signatures that support cognitive stability under physiological conditions but become saturated and destabilized under supraphysiological dopaminergic drive.

Although amphetamine-induced ISI variability could be attributed to locomotor hyperactivity, our analyses show that dmPFC calcium activity was not driven by movement speed or initiation (Fig. 3b), making this explanation unlikely. Because amphetamine elevates serotonin and norepinephrine in addition to DA, contributions from other neurotransmitter systems cannot be excluded. This “amphetamine state” mimics the neurochemical landscape of acute psychosis^39,40^, and thus, serves as a translational model for how dopaminergic overtone disrupts dmPFC population dynamics in psychosis-like states.

Single-cell–resolved imaging of the PFC was beyond the scope of the present study, which was not designed to analyze PFC microcircuit function at the level of individual neurons. Extensive prior work using single-cell imaging has already provided critical insights into PFC cellular mechanisms underlying cognition^62–65^, providing an essential foundation for the population-level dynamics examined here.

The mechanisms underlying IN-DA effects remain elusive^30^. Moreover, DA acts on multiple brain regions beyond the PFC^66^, including striatal circuits regulating working memory and cognitive control^6,67^, complicating interpretation of PFC-specific effects. Although animals were habituated, stress from drug administration and fiber-optic tethering may have contributed to the behavioral performance (Fig. 2) and cannot be fully excluded. As the spatial zone analysis (Fig. 3c) captures neural activity across a spectrum of behaviors that includes direct object exploration alongside zone entry and exit movements, interpretation of these results should be cautious. Other limiting factors may be the small sample size, the exclusive use of one sex, and the application of a single dose of IN-DA. Although calcium transients reliably reflect neuronal activity^68,69^, the neurophysiological underpinnings of fiber photometry signals remain incompletely understood. Because CaMKIIα promoter-driven viral expression can occur in a subset of GABAergic interneurons^45^, the recorded calcium signals cannot be considered exclusively glutamatergic in origin and may represent a mixture of excitatory and inhibitory neuronal activity, albeit weighted toward excitatory neurons. Additionally, this study did not directly measure dmPFC DA levels or dissect DA receptor subtype-specific mechanisms, despite their roles in PFC physiology^70^ and behavior^12,71,72^. Nevertheless, by combining pharmacology with fiber photometry recording, this study uniquely characterizes how DA tuning shapes the temporal organization of dmPFC activity in cognitively relevant states.

## Conclusion

This study provides population-level evidence that dopaminergic state shapes the temporal organization of dmPFC activity. Our findings indicate that IN-DA supports cognitive processing not merely by scaling overall neural activity, but by tuning the frequency and temporal regularity of prefrontal population events. ISI stability thus emerges as a precise, physiologically grounded index of prefrontal dopaminergic tone, and a potential therapeutic target in disorders characterized by dopaminergic dysfunction, such as ADHD and schizophrenia^73^.

## Supplementary Information

Below is the link to the electronic supplementary material.

Supplementary Material 1

## Data Availability

The datasets generated and/or analyzed during the current study are available from the corresponding author upon request.
