# Supplementary material for: Intranasal dopamine stabilizes prefrontal cortical CaMKIIα-positive neuronal population dynamics during object discrimination
Source: Sci Rep. 2026 Aug 6;16:36829. doi: 10.1038/s41598-026-65870-1 (PMC13736705; doi:10.1038/s41598-026-65870-1)
Supplement: Supplementary file 1 [file 41598_2026_65870_MOESM1_ESM.docx]

**Supplementary Figure 1.** Analysis of calcium dynamics across spatial zones. Two-way repeated ANOVA, with “drug” treatment (VEH or DA) and “zone” (object 1/old, object 2/novel, or non-object area) as within-subject factors, revealed significant main effects of “zone” on the mean z-score and number of calcium spikes during the learning trial. During the testing trial, significant “drug × zone” interactions and main effects of “zone” were observed for both measures. A significant main effect of “drug” was also detected for the number of calcium spikes during the testing trial. # significant differences versus the object zones (overall comparison; see Figure 3c); ***p*<0.01, paired *t*-test.

**Supplementary Figure 2.** Raw calcium traces aligned to entry events for individual subjects in the peri-event analyses.
